# True and Illusory Benefits of Modeling: Comment on “Genome-Scale Metabolic Network Reconstruction and In Silico Analysis of Hexanoic Acid Producing *Megasphaera elsdenii*. Microorganisms 2020, *8*, 539”

**DOI:** 10.3390/microorganisms8111742

**Published:** 2020-11-06

**Authors:** Nicolai S. Panikov

**Affiliations:** College of Sciences, Northeastern University, Boston, MA 02115, USA; n.panikov@northeastern.edu

Lee et al. [1] recently published a paper that is part of the special issue “Genome-Scale Modeling of Microorganisms in the Real World”. In this article, the authors selected *Megasphaera elsdenii* as a promising industrial producer of hexanoic acid and developed the genome-scale model that has been claimed to improve the mechanistic understanding of the metabolic control of microbial biosynthesis important for optimization of industrial bioprocess. I feel that the authors underestimated several remarkable features of *Megasphaera* that perhaps were even more intriguing and attractive than production of hexanoic acid. Besides a number of derived from in silico analysis look contradictory and worth of open discussion.

Why *Megasphaera elsdenii*? Presently, we have nearly 300,000 fully sequenced microorganisms, while genome-scale reconstructions cover no more than 100 species. Therefore, the judicious selection of organism is important. The authors justify their choice by the potential biotechnological significance of *M. elsdenii* as a producer of hexanoic acid and as a probiotic for ruminant animals. Yet, there are several other missed features of this bacterium, which are probably even more important. Let us start from the name. The genus name *Megasphaera* stands for big spheres. Indeed, these bacteria are real giants among prokaryotes with their 2.6 m coccoid cells organized into characteristic chains up to 20 m and longer (Figure 1). It makes *M. elsdenii* a very attractive model organism for in vivo and in situ studies; their cells can be easily identified with optical microscopy even in communal specimens due to their distinctive morphology and especially in combination with selective staining (immune-fluorescence, FISH). The specific epithet *elsdenii* came after the name of the famous British microbiologist Sidney Elsden. He not only isolated and described these bacteria but also contributed to the fundamentals of metabolic reconstruction, which is the central focus of this Special Issue. In particular, he introduced the concept of molar yield, Y_ATP_ [2], which links microbial growth with metabolic stoichiometry.

*M. elsdenii* is traditionally called a rumen organism [4,5]; however, recently, it was also discovered as a permanent commensal in the human microbiome inhabiting the oral cavity, digestive tract, and vaginal tract of humans [6,7,8]. Furthermore, several *Megasphaera* strains were found to have strong immunomodulation or/and neuroprotective activities [9]; that is why they are currently engaged in development of the anticancer bacteriotherapy and for treatment of neurodegenerative diseases such as Parkinson’s and Alzheimer’s. Some *Megasphaera* are a kind of “misdemeanor”; e.g., *M. cerevisiae* is known as the bottled beer spoiler.

Apart from highly promising practical applications, *M. elsdenii* has attracted the attention of microbiologists by its unique structural and metabolic properties; some of them are listed below:

A. The bacteria have a unique pseudo-outer membrane, making their cells stained Gram-negative contrary to other members of the phylum *Firmicutes,* which is the largest portion of the human gut microbiome. The role of this membrane has not yet been clarified.

B. The energy-generating metabolic network of *M. elsdenii* is also unique, as it combines fermentation (substrate-level phosphorylation) with anaerobic respiration (ATP generation via the electron transport chain). The terminal electron acceptor (TEA) has been identified as acrylyl-CoA, the fermentation intermediate; in the course of anaerobic respiration, it is reduced to propionate [10]. The uniqueness of this TEA is that it belongs to the category of intermediates, being produced and instantly consumed in a metabolic network. For comparison, other known respiring anaerobes use external sources of TEA, such as nitrate, sulfate, CO_2_, Fe(III), Mn(IV), etc.

C. The C sources for *M. elsdenii* are limited to lactate (the first choice) and several sugars [11], while the spectrum of metabolic end products is rather wide, including a near-complete homologous series of primary monocarboxylic C1 to C6 fatty acids. It remains unknown which factors (stoichiometric, genetic, or environmental) control the mixed fermentation and the split of the C flow between multiple (up to seven) alternative pathways.

D. Many basic metabolic features of *M. elsdenii* are strain-dependent. The majority of other microorganisms also display variability between strains, but these qualities are mostly non-essential for growth activity such as secondary metabolism. In the case of *Megasphaera,* variability has been observed in the most essential qualities; e.g., some strains can grow in a chemically defined media with a single C source, while other require complex media with peptone and yeast extract. Some strains are stable and robust, while others are fastidious. Generally, *Megasphaera* and closely related human and ruminal commensals (*Veillonella, Dialister, Anaeroglobus, Negativicoccus*) have a speculative reputation of highly fastidious and hard to culture organisms: they display an extended and poorly predictable lag phase, frequent inoculation failures, low yield, and high mortality. The spectra of fermentation products also vary among different strains of the same species [12]. 

Thus, *M. elsdenii* is interesting not only because of its industrial potential; it also triggers research curiosity by many intriguing features. The genome-scale metabolic models (GEMs) could give a powerful impetus for a better understanding of their biology and biotech optimization. GEMs are especially attractive for “accelerated domestication” of the recently discovered microorganisms including the development of chemically defined media and resolving numerous operational issues behind growth instability [13,14,15]. Let us see now how efficiently the GEM works for *M. elsdenii,* and whether it is able to explain any of the enigmatic qualities outlined above.

The genome-scale reconstruction of *M. elsdenii* was performed by Lee et al. [1] according to the firmly established procedure [16]. It does not mean that this job was easy and straightforward. Any GEM for a new organism requires a lot of manual effort, which includes searching published records and other resources, correcting elemental and charge imbalances, matching genes to appropriate biochemical reactions, filling the gaps, correcting annotation errors, etc. The developed FBA model called *i*ME375 covers about 16% of the total genome accounting for 375 genes, 521 reactions, and 443 metabolites. It was a significant step forward. The indisputable achievement of this work is that the first carefully designed and refined FBA (Flux Balance Analysis) model for *M. elsdenii* was made available in a standard SBML (Systems Biology Markup Language) format to other potential users. Now they can download *i*ME375 and run the model for their own specific applications, add new elements, and further improve it including a higher coverage of genes and reactions. 

The experimental validation of GEM was based on a comparison of simulation with three sets of experimental data (Table 1 in [1]); the authors proudly depict these results as evidence of the “remarkably high consistency” of their *i*ME375 model authentically reproducing (i) the specific growth rate of bacteria (*μ*), (ii) specific C source (lactate or glucose) uptake rates (*q_s_*), and (iii) the rates of fermentation products formation (*q_p_*). The statement seems to be overoptimistic. Three points below specify our concern.

1. Selection of Verification Variables. We should distinguish the rates generated by a model and those used as boundary conditions, i.e., the fixed values taken from published sources. The glucose or lactate uptake rates *q_s_* belong to the second category [16]; therefore, they cannot be used for validation. The *μ-*value is also not a perfect validation test because of linear correlation with *q_s_* [17]:

(1) $$\mu =Y(q_{s}-m)\approx Yq_{s}$$

where *Y* and *m* are two very conservative biokinetic parameters, the yield and maintenance coefficient, respectively. The maintenance account is really significant only under deep substrate limitation in chemostat culture but not in the substrate-sufficient exponential growth phase of a batch culture when $q_{s}\gg m$. The growth yield *Y* (g cell mass produced per g consumed substrate) does not vary too much among diverse fermenting organisms. Thus, with the freedom to select any *q_s_*, the successful *μ* simulation is equivalent to an adequate prediction of *Y,* which is not a challenging task and not a strong validation test. Potentially only *q_p_* values, specific rates of products formation, can be used fruitfully for model verification. Unfortunately, out of six potential *M. elsdenii* products, we can see only 2–4 entries, and there was not any discussion of why the other products were missed.

2. Wrong Choice of Experimental Data Sets. The heart of FBA is the balance of mass, energy, charge, elements, etc. However, the balancing would be impossible if some consumed C-sources are completely ignored. That is what exactly happened with the validation of *i*ME375: two out of three sets of experimental data were obtained by using complex media with glucose or lactate combined with yeast extract (YE) and peptone (P), the complex component being not accounted for in the model. The authors try to convince reader that the YE and P contribute no more than “traces of amino acids” and are fully consumed by bacteria over early exponential phase. This is obviously wrong. First, P and especially YE contain a wide array of individual compounds apart from amino acids that should have been transported and metabolized via pathways distinct from those accounted for in the *i*ME375 model. Second, concentrations of YE and P were too high to be considered as ‘traces’, specifically 10 g/L each in the first experimental set versus 8.0 g/L of glucose [18] and 0.6 g/L of YP versus 3.15 g/L of lactate in the second set [19]. Finally, as was shown with *E. coli* and other microorganisms [13,14,15], in the presence of YE and P, the microbial metabolic network undergoes dramatic reconfiguration, making the FBA solutions for complex and minimal media completely different. In order to apply GEMs for auxotrophic cells that are not able to grow on minimal media, the initial conditions are formulated as a vector *q**_s_*** = [*q*_1_, *q*_2_, … *q*_n_] for consumption rates of *n* individual compounds coming from complex media [20,21,22,23,24]. The model *i*ME375 does not include such vectors; therefore, only one set of experimental data [5] can be left for further discussion. To prove a hopeless irrelevance of the complex media data, we calculated the apparent cell yield on glucose *Y* (green curve) and *μ* (blue curve) from the redrawn residual glucose and cell biomass (Figure 2, top). As expected, the apparent *Y* was absurdly high because of the non-accounted consumption of YE and P. Even by the end of the growth phase, *Y* remains 0.24 g/g, which is still too high for anaerobic growth, indicating that glucose is not the only C source.

3. Errors in Identification of Growth Parameters. Finally, the validation of *i*ME375 by using the minimal medium data also remains inconclusive. Figure 2 (bottom) shows data points redrawn from the original publication [5] with our curve calculated from the simple exponential model. We assume that *μ* is constant and the maintenance *m* = 0; then, the biomass (*x*) and residual lactate (*s*) follow two differential equations:

(2) $$\frac{dx}{dt}=\mu x\;\frac{ds}{dt}=-\frac{\mu x}{Y}$$

After integration under initial conditions *x = x*_0_, *s = s*_0_ at *t* = 0, we have two equations to be fitted to the experimental data:

(3) $$x=x_{0}e^{\mu t}\;s=s_{0}-{\frac{x_{0}}{Y}}_{0}\left(e^{\mu t}-1\right)\;$$

Non-linear regression with Microsoft Solver gives the following best-fit parameters: *q_s_* = −32 mmol/h/g cells, *μ* = 0.29 h^−1^, *Y* = 0.08 g/g. The *q_s_* value is in agreement with [1] but our *μ* estimate turned out to be ≈5 times higher: 0.29 vs. 0.06 h^−1^! Then, curiously enough, the authors find (see Figure 3 in [1]) much higher *μ*-values reaching 0.6 h^−1^! A probable reason for failure of the *i*ME375 to reproduce a correct *μ* value was the too high COBRA parameter NGAM (non-growth associated maintenance) 3.5 mmol ATP/h/g borrowed from the chemostat study [25].

The FBA model is used for better understanding of bacterial metabolism and optimization. Sadly, none of the points A through D in our introductory sections were touched. Basically, it was possible to clarify point C about control of the mixed fermentation, but paper [1] misses this data and discussion, focusing only on hexanoic acid. Point B (unusual anaerobic respiration) also has been left without an explicit presentation of corresponding fluxes. Instead, the major attention was paid to a fragment of the whole metabolic network representing the diverged branched pathway called the *bifurcated hexanoic acid synthetic pathways*. To get a mechanistic insight into how the splitting of the C flow is regulated, the authors applied the *flux ratio analysis*. They run *i*ME375 with maximizing hexanoic acid production as an objective function under a fixed glucose uptake rate 5 mmol/h/g and constraining microbial growth rate at a series of values. The result was (see Figure 3 in [1]) that the canonic pathway via acetyl-CoA (route A, red color code) was inversely related to the growth rate, while a more exotic reversed TCA cycle route B (blue) stayed nearly the same at all tested *μ*. In the second computational experiment, the split ratio between routes A and B was forcibly varied at each *μ*, and it did not affect the simulated hexanoate production very much. It gave the ground for the principal conclusion of this study that the highest hexanoic acid production is achieved with “the balanced fractional contribution” of two pathways. The meaning of the word *balanced* remains unclear, but the undertaken in silico approach eventually led to the pessimistic conclusion that genetic manipulations (knockdown/overexpression) of the enzymes *pfo* and *pyc* next to the branching point are likely not able to improve the productivity of the strain.

I believe that the presented results are not ready for any recommendation related to the practical metabolic engineering and fully agree with the authors stating that “a comprehensive understanding of the … in silico strain design is needed”. To explain the simulation results, we address the revised version of the branching pathway (Figure 3). There are two competing pathways A (red) and B (blue) extended to the point where they merge, producing the crotonyl-CoA that is finally converted to the end product hexanoate. I also added the variable missed by the authors, it is the biomass, which is linked globally to all intracellular metabolites. Indeed, the metabolites’ flow from pyruvate to hexanoate is not isolated from the rest of the metabolic network, leading eventually to cellular reproduction; all of the intermediates, although in different degrees, are diverted to biomass synthesis via selected precursors identified in the half-empirical “biomass reaction”. Taking into account the withdrawal of metabolites for biomass synthesis, we conclude that there are at least three rather than two processes that compete with each other: route A, route B and biomass production with the sum of routes A and B. However, how do we simulate the *natural* regulatory way for splitting three metabolic flows? The conventional FBA way is to use the biomass formation as an objective function and then apply linear programming to find such a fluxes pattern that maximizes *μ*. The tiny fragment of this pattern, the fluxes immediately downstream of the branching points, will inform us about the partition of the A and B routes, supporting the fastest growth.

To explore the true natural relationship between the growth rate and hexanoate production, we should start from the question: what are experimental ways to modulate bacterial growth rate? We have several experimental options: (i) running a series of batch cultivations with different C sources, each providing a unique maximum specific growth rate, (ii) using non-lethal growth inhibitors at a series of concentrations, and (iii) running chemostat culture at different dilution rates. The last approach is the best if not the only reasonable option in combination with GEMs. Fortunately, even experimental chemostat data are available for *M. elsdenii* [25]; we need only minor FBA modifications to set up *μ* dependence on a limiting substrate concentration such as a Monod equation or more advanced models. It can be done in the future, but presently, the approach used in [1] does not work: in real life, growth deceleration (decrease of *μ*) is always accompanied by a corresponding decrease of *q_s_* (see Equation (1)), while the authors dramatically changed *μ* at the constant glucose uptake rate. The C balance for this particular situation looks as follows:

(4) $$\begin{array}{c} \mathrm{growth}\\ \mu x \end{array}+\begin{array}{c} \mathrm{products}\;\mathrm{formation}\\ q_{p}x \end{array}=\begin{array}{c} C\;\mathrm{source}\;\mathrm{uptake}\\ q_{s}x \end{array}=constant.$$

The sum of two terms for growth and products formation is kept constant; hence, growth restriction should produce an equivalent increase of the product’s formation. It explains the intensification of route A at a lower growth rate (see Figure 3b in [1]). The pattern for route B is less clear; formally, hexanoic acid production via route B is not coupled with cell biosynthesis, but it can be just an artefactual simulation result. Probably, an account of other fermentation products apart from hexanoic acid can bring further clarification.

Concluding our review, the interpretive value of the *i*ME375 seems not very high; the conclusions generated by the flux ratio analysis seem to be not convincing and should not be recommended for immediate practical implementations. The criticism should not be taken as discouragement. Development of GEMs and their practical applications belong to a novel area where every new step is not easy but highly valuable. The paper [1] definitely combines valuable result manifested as true modeling benefits with a number of less productive in silico simulations that presently do not add too much the microbiological knowledge. However, even ‘playing’ with a GEM model by applying it to an unrealistic problem far away from biotechnological needs is not a wasting of time! It provides a useful training exercise to learn one of the most powerful computational tools for the modern bioindustry. 

## Figures and Tables

**Figure 1:**
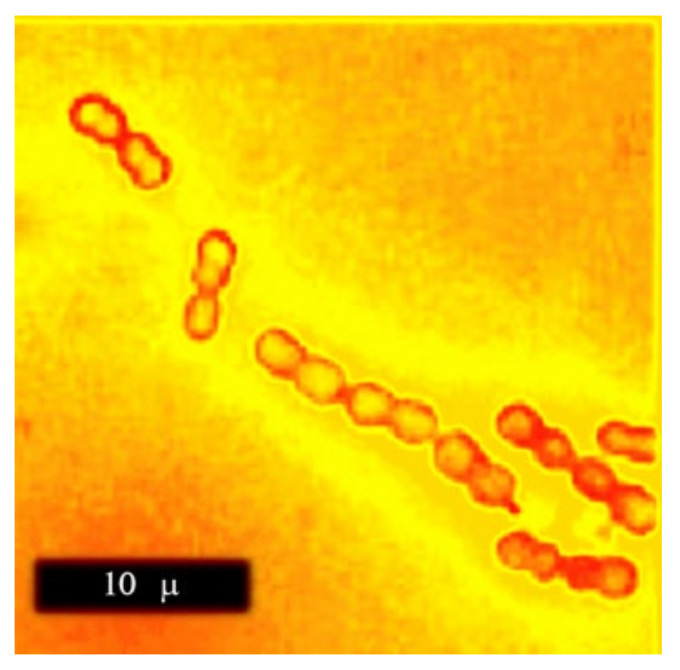
*M. elsdenii* cells, reproduced from [3] with permission.

**Figure 2:**
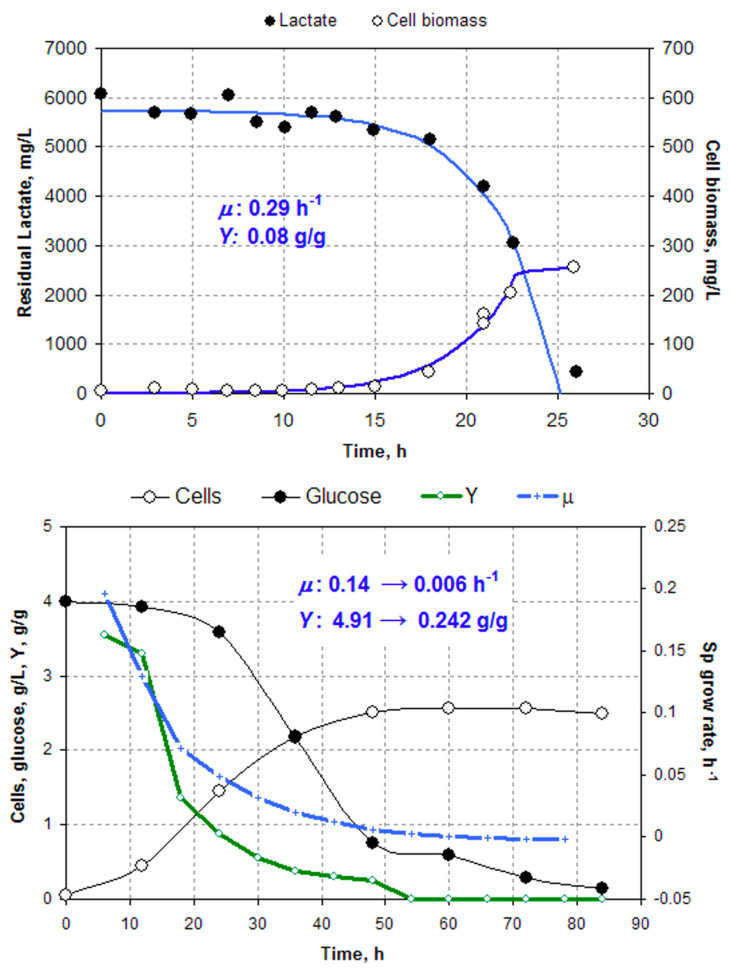
Experimental validation of FBA. Top: *M. elsdenii* growth on complex medium [18]. Bottom: the minimal medium with lactate [5]. Curves were calculated from Equation (3).

**Figure 3:**
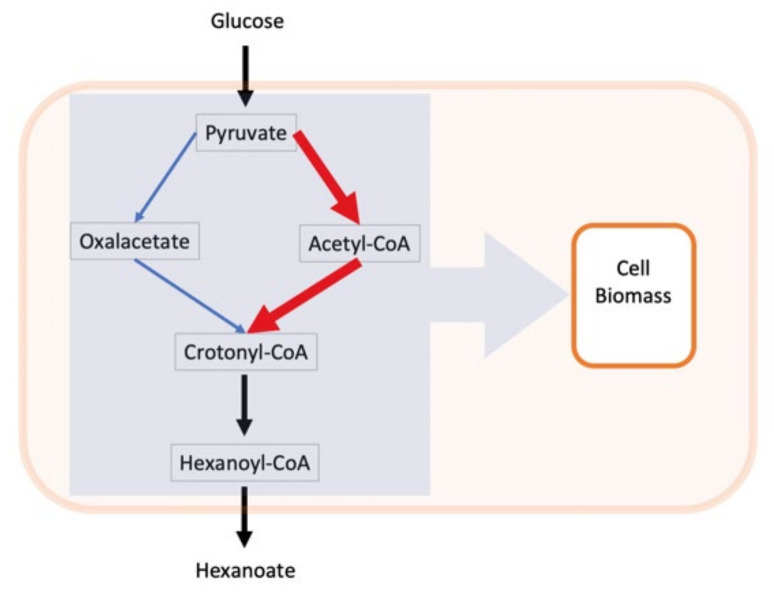
Schematic representation of the branched pathway as related to bacterial growth rate. The orange frame stands for the cell wall separating the intracellular and extracellular variables.
